# Automatic Electronic Laboratory-Based Reporting of Notifiable Infectious Diseases

**DOI:** 10.3201/eid0807.010493

**Published:** 2002-07

**Authors:** Anil A. Panackal, Nkuchia M. M’ikanatha, Fu-Chiang Tsui, Joan McMahon, Michael M. Wagner, Bruce W. Dixon, Juan Zubieta, Maureen Phelan, Sara Mirza, Juliette Morgan, Daniel Jernigan, A. William Pasculle, James T. Rankin, Rana A. Hajjeh, Lee H. Harrison

**Affiliations:** * Centers for Disease Control and Prevention, Atlanta, Georgia, USA; † Pennsylvania Department of Health, Harrisburg, Pennsylvania, USA; ‡ University of Pittsburgh, Pittsburgh, Pennsylvania, USA; § Allegheny County Health Department, Pittsburgh, Pennsylvania, USA

**Keywords:** bioterrorism, electronic laboratory-based reporting, Health Level 7 (HL7), real-time, capture-recapture, National Electronic Disease Surveillance System (NEDSS)

## Abstract

Electronic laboratory-based reporting, developed by the University of Pittsburgh Medical Center (UPMC) Health System, was evaluated to determine if it could be integrated into the conventional paper-based reporting system. We reviewed reports of 10 infectious diseases from 8 UPMC hospitals that reported to the Allegheny County Health Department in southwestern Pennsylvania during January 1–November 26, 2000. Electronic reports were received a median of 4 days earlier than conventional reports. The completeness of reporting was 74% (95% confidence interval [CI] 66% to 81%) for the electronic laboratory-based reporting and 65% (95% CI 57% to 73%) for the conventional paper-based reporting system (p>0.05). Most reports (88%) missed by electronic laboratory-based reporting were caused by using free text. Automatic reporting was more rapid and as complete as conventional reporting. Using standardized coding and minimizing free text usage will increase the completeness of electronic laboratory-based reporting.

Public health surveillance of infectious diseases is crucial for detecting and responding to illnesses that may represent potential outbreaks or bioterrorism events (1,2). The Centers for Disease Control and Prevention (CDC) is collaborating with state health departments to improve current disease surveillance by using a standards-based information architecture through the National Electronic Disease Surveillance System (NEDSS), which includes electronic laboratory-based reporting of certain diseases to local, state, and federal public health authorities (3–5). Automatic reporting at private clinical laboratories in Hawaii has been shown to be more rapid and complete than conventional reporting (6).

Allegheny County (population 1,348,000) is located in southwestern Pennsylvania and includes the city of Pittsburgh. Incidences of notifiable diseases in the county are required by law to be reported directly to the Allegheny County Health Department (ACHD). Each notifiable event is recorded on a case report form that is mailed or faxed to the ACHD by laboratory personnel, physicians, nurses, or infection-control staff; this procedure constitutes the conventional paper-based reporting system (Figure 1). A notifiable event is considered reported when received and confirmed by the health department.

**Figure 1:**
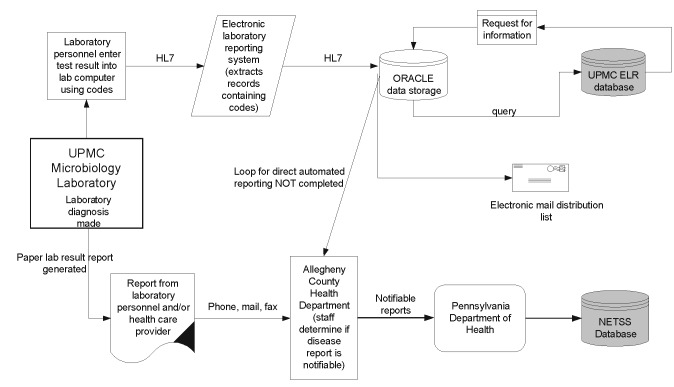
Schematic of information flow for the electronic reporting system of the University of Pittsburgh Medical Center Health System and for the paper-based reporting system to Allegheny County Health Department. NETSS, National Electronic Telecommunications System of Surveillance. ELR is electronic laboratory-based reporting, and CRS is conventional paper-based reporting system.

The UPMC Health System is a large university-based health-care network consisting of approximately 20 hospitals and hospital affiliates (http://www.upmc.edu) in western Pennsylvania and is affiliated with the University of Pittsburgh School of the Health Sciences. UPMC established real-time, electronic laboratory-based reporting. This system is based on an existing hospital communications infrastructure designed to improve the speed and completeness of reporting (Wagner MM et al., unpub. data). ACHD personnel estimate that 40% of all notifiable infectious diseases reported to the ACHD come from UPMC. We evaluated the accuracy, completeness of coverage, and timeliness of electronic laboratory-based reporting before the integration of electronic laboratory-based reporting into the conventional paper-based reporting system (7).

## Background

Eight UPMC hospital microbiology laboratories in Allegheny County are capable of electronic laboratory-based reporting by using Health Level 7 (HL7), an electronic messaging standard for data exchange and communication between health-care information systems (http://www.h17.org). Laboratory personnel and health-care providers who obtained results from these laboratories were required to report through the paper-based system and were unaware of the establishment of new electronic reporting (Figure 1). Once a laboratory technician obtained a test result, he or she entered the information into the hospital laboratory computer, which generated an HL7 message. Although laboratory workers could enter test results by using preprogrammed codes or free text (non-coded, non-standardized text entered by a laboratory personnel), the electronic laboratory-based reporting system monitored only coded organism names in each HL7 culture message.

The processing occurred in real time, i.e., messages were checked as they were received. Instead of a batch mode in which data are extracted from sets of reports at predetermined times, extraction of information occurred whenever data were received by the electronic system. The electronic system extracted the specific laboratory specimen, procedure, and result from the HL7 records. The data were then interpreted; a laboratory result was positive or negative based on the code in the result section, which was compared with a data dictionary developed by the UPMC Health System. Some duplicate records were recognized by the electronic system. The extracted three- or four-letter coded organism name, defined by the UPMC laboratory system data dictionary, was then converted to its full name through a translation table maintained in the Oracle (Oracle Corporation, Redwood Shores, CA) data storage; computer personnel could use the database to add or remove an organism they wanted to be monitored. To obtain complete patient demographic information, if not provided in the laboratory HL7 message, the electronic laboratory-based reporting system queried the Medical Archival Retrieval System at UPMC based on the patient’s medical record number in the message (8). Simultaneously, an electronic mail message containing the laboratory test result was sent by the electronic reporting system to selected UPMC personnel. Of note, the loop for direct automatic reporting between UPMC and ACHD was not completed at the time of this evaluation and, thus, no notifiable events were reported by the electronic system to the ACHD.

## Methods

We conducted a comparison evaluation of the UPMC electronic laboratory-based reporting and the ACHD conventional paper-based reporting systems. From the eight UPMC hospital or affiliated microbiology laboratories with HL7 links in Allegheny County, we compared all disease reports in the UPMC electronic and the ACHD paper-based systems (derived from the National Electronic Telecommunications System of Surveillance) databases with dates of positive culture from January 1 to November 26, 2000, for 10 infectious organisms: *Campylobacter*, *Cryptosporidium*, *Escherichia coli* O157:H7, *Giardia*, *Listeria*, *Legionella, Neisseria meningitidis*, *Salmonella*, *Shigella*, and *Yersinia*. The diseases caused by these organisms are notifiable to ACHD, requiring specific laboratory findings to meet the CDC case definition for notifiable diseases (9,10). Reporting of *Legionella* was evaluated for the period June 21–November 26, 2000, because the UPMC electronic laboratory-based reporting did not capture reports of diseases caused by this organism before June 21. Duplicate records and cultures performed in the context of research studies not notifiable to ACHD but included in the UPMC electronic database were excluded. Case reports in each database were matched manually by the investigator. A match was defined as a report in the UPMC electronic database that had the same patient name, date of birth, and type of notifiable infectious disease as a report in the ACHD paper-based database. After matching, the case reports that were found in both databases, as well as cases found in only one of the two databases, were entered into a separate Excel (Microsoft Corp., Redmond, WA) spreadsheet.

**Figure 2:**
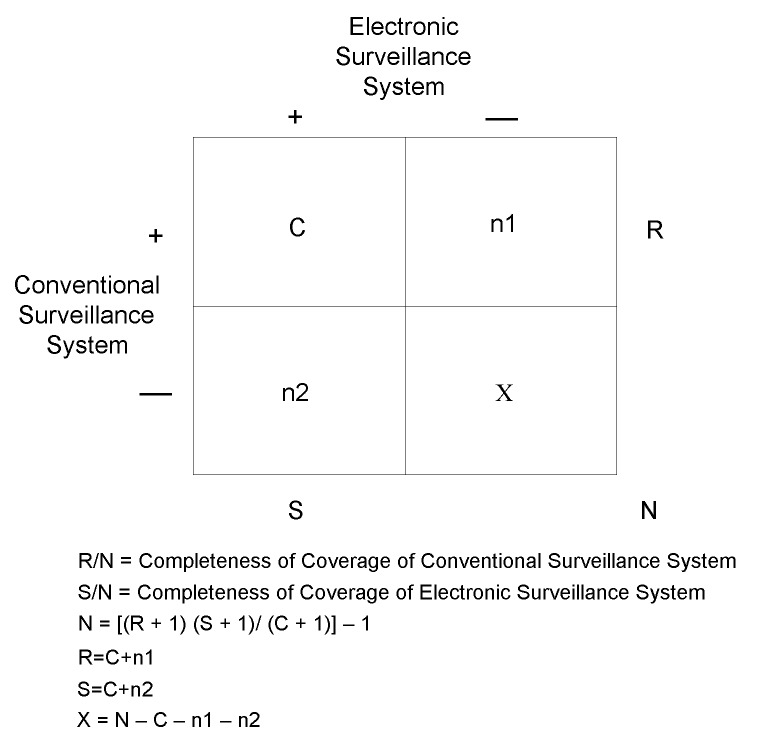
Capture-recapture methodology (11). C=number of reports received through both electronic laboratory-based reporting and conventional paper-based reporting. n1=number of reports received through conventional paper-based reporting system only. n2=number of reports received through electronic laboratory-based reporting only. X= estimated number of reports missed by both electronic laboratory-based reporting and conventional paper-based reporting system. R=number of reports received through conventional paper-based reporting system. S=number of reports received through electronic laboratory-based reporting. N=estimated total number of reports available by the Chandra Sekar-Deming capture-recapture calculation.

Completeness of reporting was defined as the total number of unique, notifiable events identified independently through each surveillance system (UPMC electronic laboratory-based and ACHD conventional paper-based systems) divided by the estimated total number of reports available for reporting at the laboratory level (N) (Figure 2). To estimate the total number of reports available, we used the Chandra Sekar-Deming capture-recapture method (12). Since both the UPMC electronic and ACHD systems may not have captured all notifiable events, capture-recapture provided an approximation of the true total number of notifiable cases based on samples from these two independent, parallel surveillance systems (12,13). The overall completeness of reporting, the completeness of reporting by disease and by hospital, and the 95% confidence interval (CI) for completeness of coverage calculations were determined by using a resampling analysis based on the capture-recapture method (11). To date, no methods for the calculation of the 95% CI for completeness of coverage have been published. When SAS version 8.1 (SAS Institute, Inc., Cary, NC) was used, the resampling was based on the assumption that the distribution of the data observed for the three cells (e.g., C, n1, n2) of the contingency table followed a uniform distribution; the 5% and 95% values of the distribution from this analysis yielded the 95% CI for completeness. Completeness could not be calculated for diseases and hospital laboratories with zero values in the 2 by 2 contingency table cells (Figure 2).

An electronic false-positive result was defined as a case that was incorrectly detected by the electronic system; a missed report (or false negative) was defined as a notifiable case that was not detected through the electronic system. The completeness of reporting of both systems was estimated after excluding false positives and duplicate reports.

**Figure 3:**
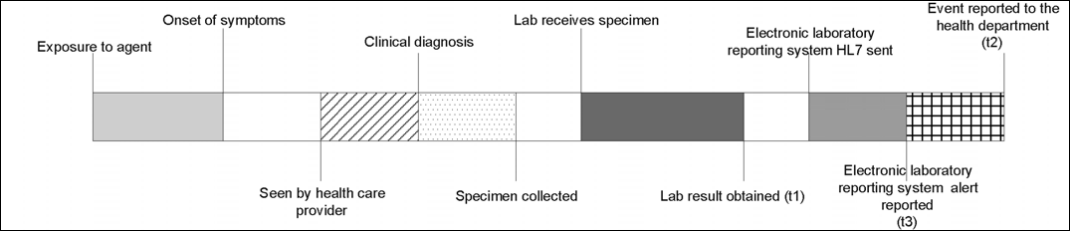
Timeline for reporting notifiable infectious diseases by the University of Pittsburgh Medical Center Health System, Allegheny County, Pennsylvania.

The chronologic sequence of events for the reporting of an infectious disease or condition consists of exposure to an infectious agent, followed by symptom onset after an incubation period, and then the seeking of medical attention (Figure 3). Although a presumptive diagnosis could be made by interpretation of the clinical syndrome at this point, the ability of electronic laboratory-based reporting system to detect a notifiable disease or condition begins at the time the laboratory result has been entered into the data system.

To determine the timeliness of the two surveillance systems, three time points were defined. T_1_ was the date/time when the laboratory result was obtained and entered into the UPMC laboratory computer. T_2_ was the date/time when the laboratory result was reported to ACHD by the conventional paper-based system. T_3_ was the date/time the automatic electronic laboratory-based system notification was generated at UPMC. The timeliness of electronic and paper-based systems was defined as t_3_ – t_1_ and t_2_ – t_1_, respectively. The difference between t_3_ and t_2_ represented how much sooner or later the electronic system identified notifiable diseases than the paper-based system. Median differences were expressed with an interquartile range. The timeliness calculations were performed with SAS version 8.1.

Before matching individual records in both systems and removing duplicate records, we calculated the completion rates for the data fields common to both the UPMC electronic and the ACHD paper-based databases by using Epi-Info 2000 (Centers for Disease Control and Prevention, Atlanta, GA).

We identified the specific reasons for the electronic system’s false positives and missed reports by using a traceback error analysis. Reports that were found in the UPMC electronic database but not in the ACHD paper-based database were identified, and case-patient information was reviewed from the laboratory computer reports and their HL7 messages (electronic false positives). Reports that were found in the ACHD paper-based database and not in the UPMC electronic database were identified after reviewing case-patient information, laboratory case reports, and archived computer files (electronic laboratory-based reporting missed reports). To further assess database accuracy, we also reviewed the paper reports and logs at the Allegheny County Health Department and compared these with data in the ACHD conventional paper-based reporting system database.

## Results

**Figure 4:**
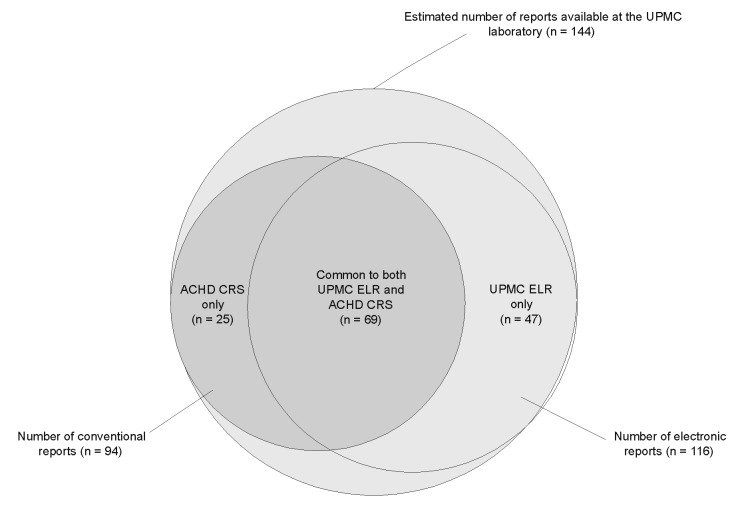
Venn Diagram depicting the number of notifiable disease reports received independently by the electronic laboratory-based reporting of University of Pittsburgh Medical Center electronic system, Allegheny County Health Department paper-based reporting, or both. The estimated true total number of reports available, calculated by the Chandra Sekar-Deming capture-recapture method, is shown in the large, encompassing circle. ELR is electronic laboratory-based reporting, and CRS is conventional paper-based reporting system.

A total of 141 unique reports were identified; 116 (82%) were reported through the UPMC electronic laboratory-based system, and 94 (67%) were reported by ACHD conventional paper-based reporting system. Forty-seven (33%) of the notifications were received through the UPMC electronic system only, 25 (18%) through the ACHD paper-based system only, and 69 (49%) through both (Figure 4). The estimated total number of reports calculated by the capture-recapture method was 144.

After excluding electronic laboratory-based reporting false positives, the overall completeness of reporting was 74% (95% CI 66% to 81%) for the UPMC electronic system and 65% (95% CI 57 to 73%) for the ACHD paper-based system (p>0.05), showing no significant difference in completeness of reporting between the electronic and paper-based systems (Table 1). Table 1 also lists the completeness of coverage and 95% CI by disease and by hospital. Most of the cases missed by electronic reporting were from one hospital (UPMC Hospital C).

**Table 1:** Completeness of coverage for UPMC electronic and conventional reporting systems by the notifiable infectious disease and hospital laboratory^a^

		Conventional reporting (ACHD)		Electronic reporting (UPMC)	
	Total no. of available reports^b^	No. of reports received	Completeness of coverage (95% CI)	No. of reports received	Completeness of coverage (95% CI)
Notifiable infectious disease					
*Campylobacter*	37	25	0.68 (0.49 to 0.85)	18	0.49 (0.32 to 0.65)
*Salmonella*	35	32	0.91 (0.83 to 0.97)	34	0.95 (0.91 to 0.97)
*Escherichia coli* O157:H7	17	10	0.59 (0.33 to 0.86)	7	0.41 (0.19 to 0.67)
*Giardia*	22	13	0.59 (0.39 to 0.77)	17	0.77 (0.58 to 0.90)
*Neisseria meningitidis*	9	5	0.58 (0.30 to 0.88)	7	0.72 (0.46 to 0.88)
					
UPMC Hospital laboratory					
A	26	16	0.62 (0.46 to 0.80)	24	0.92 (0.81 to 0.96)
B	52	29	0.55 (0.42 to 0.65)	47	0.91 (0.79 to 0.96)
C	35	24	0.69 (0.43 to 0.90)	9	0.26 (0.12 to 0.40)
D	13	11	0.85 (0.64 to 0.92)	12	0.87 (0.71 to 0.92)
E	10	9	0.90 (0.70 to 0.90)	9	0.90 (0.70 to 0.90)

^a^UPMC, University of Pittsburgh Medical Center Health System; ACHD, Allegheny County Health Department; CI, confidence interval. ^b^Estimated total number of reports available by using capture-recapture (N in Figure 2).

Timeliness was calculated by using the 69 records common to both databases. The timeliness of paper-based reporting was a median of 5 days (interquartile range 4 days). The timeliness of the electronic reporting was a median of 1 day (interquartile range 0 days). Electronic alerts were reported a median of 4 days (interquartile range 4 days) sooner than through paper-based reporting. We discovered a trend in the UPMC electronic reporting: the time difference between the date/time the laboratory result was obtained and entered, and the date/time the HL7 message was sent was almost exactly 24 hours to the second. After extensive discussions with the UPMC laboratory administration and informatics personnel, the reasons for this finding are unknown.

Eleven data fields were common to both the UPMC electronic and the ACHD paper-based databases (Table 2). Of these, six fields were 100% complete in both. Of the remaining five, two were more complete in the UPMC electronic system (date of birth and age), whereas three were more complete in the ACHD paper-based system (address, zip code, report status [i.e. final results]); these differences were significant (p<0.001).

**Table 2:** Data field completion rates on common data fields for cases in UPMC electronic and conventional reporting system databases^a^

Data field	No. (%) of conventional reported cases with field completed (n=534)	No. (%) of electronic reported cases with field completed (n=582)
Patient information		
Patient ID	534 (100)	582 (100)
Name	534 (100)	582 (100)
Sex	534 (100)	582 (100)
Date of birth	462 (86.5)	582 (100)
Age	518 (97.0)	582 (100)
Address	533 (99.8)	306 (52.6)
Zip code	533 (99.8)	306 (52.6)
		
Specimen information		
Organism name	534 (100)	582 (100)
Time result obtained	534 (100)	582 (100)
Time result reported	534 (100)	582 (100)
		
Other information		
Status of report	534 (100)	220 (37.8)

^a^All rates before matching and duplicate record removal; UPMC, University of Pittsburgh Medical Center Health System.

Electronic laboratory-based reporting generated 10 reports that were found to be false upon investigation (Table 3). Since the electronic reporting system can only capture diseases that are entered with the preprogrammed UPMC disease codes, test results entered with free text could not be extracted correctly into the UPMC electronic database. Most of the identified errors were, in fact, caused by the use of free text in combination with the UPMC code for the organism (i.e., the combining of the free text “No” with an organism code when laboratory technicians entered results). For example, a result entered by laboratory technicians as “no” “SALM” (the UPMC code for *Salmonella*) was recognized and incorrectly detected by electronic system as a positive case of *Salmonella*. Other errors involved the inability to retract preliminary reports of the isolation of notifiable organisms that were not subsequently confirmed and data extraction from the incorrect part of the result field. In the latter instance, a case of legionellosis was reported as listeriosis because the data field included the phrase “Specimen Delivery: UNIT LIST,” and LIST is the disease code at UPMC for *Listeria*.

**Table 3:** Electronic false positives and missed reports in UPMC reporting system^a^

Errors	No. (%) of electronic or paper-based only reports	Nature of problem
Electronic false positives		
Incorrect use of free text with organism codes	6 (60)	Culture report reads “No [free text]” followed by organism ID code
Inability to retrieve sent false reports	3 (30)	Unable to retrieve preliminary reports
Failure of logic detection	1 (10)	Data extracted from wrong portion of result field by logic detection
Total	10	
Electronic false negatives (missed reports)		
Incorrect use of free text	22 (88)	Organism name typed out as free text in result field
Unknown (failure of transmission?)	3 (12)	Found to be in UPMC hospital computer terminal system by using organism ID code properly but not found in UPMC electronic database
Total	25	

^a^UPMC, University of Pittsburgh Medical Center Health System.

Data-entry errors, such as the incorrect use of free text, led to missed reports in the electronic system. Typically, these errors occurred when laboratory technicians entered the name of the organism as free text rather than with the preprogrammed UPMC disease codes. These errors accounted for 22 (88%) of 25 electronic missed reports, whereas the remaining three missed reports were found in the hospital computer systems but were not detected by the UPMC electronic system for reasons that remained unclear after investigation. Of the 47 cases in the UPMC electronic system not reported by the paper-based system to ACHD, 37 should have been reported to ACHD (“ACHD false negative”).

## Discussion

This evaluation is the first of a large health-care system by using automatic and electronic notifiable disease reporting. The electronic laboratory-based reporting was as complete as conventional paper-based reporting. The estimated completeness (74%) is similar to the recent report of 80% completeness of the electronic laboratory-based reporting from commercial clinical laboratories to the Hawaii Department of Health (6). The incompleteness and inaccuracy of UPMC electronic reporting was caused mainly by the use of free text, rather than standardized organism codes, by laboratory personnel at one hospital. Similarly, most of the electronic false positives were caused by the use of free text.

The magnitude of the difference in completeness between electronic laboratory-based reporting and conventional paper-based reporting may have been greater if it had been possible to review reports coming exclusively from laboratories to the ACHD; paper-based systems receive reports from sources other than laboratories. Data specifying if a case record originated from a laboratory or health-care provider were not available in the paper-based database. Hence, a bias favoring completeness of reporting by the paper-based system existed in our analysis. However, most reports received by health departments originate from clinical laboratories (14). The capture-recapture method used to calculate completeness required that the two surveillance systems (UPMC electronic laboratory-based reporting and ACHD conventional paper-based reporting system) operate independently. However, some interaction between the systems existed; the laboratory director used the generated electronic e-mail message, containing the laboratory test results, to check for potential false positives before a report was falsely sent conventionally to ACHD. This interaction was thought to be minimal (Figure 1). Other capture-recapture assumptions, such as the surveillance being performed on a stable population and only true matches and events being identified by the systems, were fulfilled (12,13).

Maximizing electronic laboratory-based reporting sensitivity is important for detecting diseases, while maximizing specificity enhances the likelihood that cases are reported correctly. Theoretically, electronic reporting has the potential to be both sensitive and specific, with few false negatives and false positives. The specificity of electronic reporting could be particularly high for diseases diagnosed by laboratory tests with a low rate of false positives (e.g., culture for enteric organisms); the diseases caused by the organisms used in this study met this qualification. Notifiable diseases based on other types of tests (e.g., serology for syphilis) would require clinical criteria to enhance specificity (information not available by electronic reporting). In this evaluation, we found that the inability to retract preliminary positive laboratory reports that were subsequently confirmed to be negative reduced the specificity of electronic reporting. However, in some instances, the benefit of early detection might supersede an occasional false-positive report. For example, early detection is paramount for some organisms, such as *Bacillus anthracis,* could represent a potential bioterrorist event. Nonetheless, a substantial amount of public health effort might be expended unnecessarily if such a laboratory finding were found to be a false positive. One must balance the tradeoffs between sensitivity, specificity, and timeliness when deciding to allow these preliminary laboratory results to be reported.

The UPMC electronic reporting has the potential to serve as a prototype for use nationally because it uses hospital-based laboratory information systems already in place to capture cases of disease that may be representative of the population at large. However, several findings from our analysis have implications for large health systems attempting to establish electronic laboratory-based reporting. The use of standardized disease codes should be encouraged because it maximizes both the sensitivity and specificity of electronic laboratory-based reporting. At UPMC, the incorrect use of free text at a single hospital substantially reduced the overall completeness of electronic laboratory-based reporting reporting. However, eliminating the use of free text may not be desirable from a laboratory personnel standpoint. As such, training laboratory personnel in the correct use of free text is important (15). Moreover, UPMC computer personnel could relegate the free text option only to a note field that provides useful information to health-care providers without generating a report; in this regard, a properly constructed result code entered through a correctly designed data entry method would be useful. The use of standardized codes has broad implications for electronic laboratory-based reporting in general. To effectively enhance the unification of NEDSS, CDC recommends the implementation of standardized coding schemes for disease names (Systematized Nomenclature of Medicine [SNOMED]; http://www.snomed.org) and laboratory test names (Logical Observation Identifier, Names, and Codes [LOINC]) (http://www.regenstrief.org//loinc/). Unfortunately, UPMC and many other health centers are not using standardized coding schemes. The lack of standardized coding requires the creation of a translation table, a process that requires refinements to maximize accuracy and completeness.

A mechanism for retracting of preliminary reports not subsequently confirmed is essential to reduce false-positive reports. Retraction is the ability to both remove an incorrect or preliminary report from the database as well as to notify the recipients of the information of the change. If the sending system does not explicitly label the message as a correction or a retraction, then the electronic laboratory-based reporting system must have logic to detect it. The detection logic simply compares the previously reported preliminary reports in a cached table with a new one. If the logic finds a match but the new report does not have any notifiable organisms, the logic will send a retraction alert to officials at the local health department or hospital laboratory administrators and remove the false-positive report from the cached table. At the time of this evaluation, such retraction capability did not exist at UPMC because the UPMC laboratory sending system did not explicitly label the message as a correction or a retraction. In the future, one option may be to label preliminary reports as “preliminary” or “suspect.” Currently, two authors have been working on the retraction capability and expect to have such functionality available soon. However, the risk versus benefit of reporting preliminary laboratory results should be weighed in making the decision to retract such reports. The best approach might be to report preliminary results for diseases that require immediate notification, while reporting confirmed results for others.

Decisions to remove certain duplicate records that were not detected by electronic laboratory-based reporting should be made before integration of automatic reporting to ACHD. Caution should be used in the removal of some type of duplicates, as this decision may need to be disease specific. For example, repeated positive sputum cultures for tuberculosis from a patient who has received the recommended course of therapy may represent persistent, active infection and drug resistance, both of which are substantial public health concerns.

UPMC electronic laboratory-based reporting had lower completion rates of data fields with important contact information (specifically, address and zip code fields) compared with ACHD conventional paper-based reporting system, which may hamper efforts by public health personnel to contact patients quickly. The implementation of automatic demographic data extraction by electronic laboratory-based reporting from other resources such as epidemiologic or administrative databases (e.g., billing records), could substantially improve the data field completion rate for electronic laboratory-based reporting.

UPMC should continue to refine its electronic laboratory-based reporting before implementing direct automatic reporting to ACHD. Electronic laboratory-based reporting should not replace conventional reporting as observations made by astute clinicians are valuable in the timely reporting of certain notifiable syndromic illnesses (Figure 3). Instead, electronic laboratory-based reporting should become integrated with and complement the existing conventional reporting system to ensure the most complete capture of notifiable disease events.

The findings from this evaluation indicate that direct automatic reporting from a health system is feasible and as complete but more rapid than conventional reporting. An error analysis showed many correctable problems; better control of the use of free text and an ability to retract preliminary reports were key areas for improvement. Standard coding schemes should be used. Health departments need to evaluate electronic surveillance systems before integrating the data into existing reporting systems. CDC and state health departments should collaborate to develop a consensus on the goals for an electronic laboratory-based reporting system intended for public health laboratory-based disease reporting. Once these goals have been determined, guidelines may be created that would assess if the system achieves these desired goals. This evaluation may be used by health departments in evaluating other electronic surveillance systems.
